# Supplementary material for: Active Case Finding for Tuberculosis in India: A Syntheses of Activities and Outcomes Reported by the National Tuberculosis Elimination Programme
Source: Trop Med Infect Dis. 2021 Nov 30;6(4):206. doi: 10.3390/tropicalmed6040206 (PMC8705069; doi:10.3390/tropicalmed6040206)
Supplement: Supplementary file 1 [file tropicalmed-06-00206-s001.zip › Supplementary- Box S1 Data proforma.pdf]

Box S1. DATA PROFORMA FOR TUBERCULOSIS ACTIVE CASE FINDING (ACF).

(Please note: Please fill up one proforma for each activity, if your organisation had undertaken multiple activities)

Details reported by: **Date :** dd/mm/yyyy

**Study ID:**			(*This will be assigned by the study investigators*)
**S.No.**	**Particulars**	**Details**	**Instructions**
**Description of ACF activity**			
1.	Name of your organization		Please give the name of the organisation. If it is State TB office of Karnataka, the name of the organisation would be 'STO-Karnataka'
2.	Funding support		Please specify who provided the funding support for the ACF activity. For example, the Axshya Samvad activity of the Union is supported by the 'Global Fund'
3.	Name of the State where activity was conducted		Provide the list of the states of India where the ACF activity is being conducted by your organisation or the unit
4.	Month and year of starting the activity		Please specifiy the date when the ACF activity was initiated by the organisation.
5.	Frequency of activity in an year	☐ Monthly<br>☐ Bi-monthly<br>☐ Quarterly<br>☐ Throughout the year<br>☐ Others:__________________	Please specify how often the activity was conducted in a year by the organisation
6.	Month and year of closing the activity		Please specifiy the date when the ACF activity was closed by the organisation. In case the activity is still ongoing, kindly indicate as still ongoing
7.	Duration of the activity in days		Kindly denote how many days of ACF activity has been conducted since the start of activity
8.	Activity settings	☐ Urban Slum<br>☐ Urban non-slum<br>☐ Rural area<br>☐ Hard-to-reach areas (including tribal hamlets)<br>☐ Others:_______________	Please tick where the activity was conducted. Tick all the relevant settings.
9.	Target population	☐ General Public in the above mentioned setting<br>☐ Tribals<br>☐ Migrants<br>☐ Refugees<br>☐ Drug users<br>☐ Household TB contacts<br>☐ Children<br>☐ Elderly<br>☐ Others:_______________	Please tick among who the ACF activity was conducted. Tick all the relevant groups.
10.	Basis for selection of area and/or population		Please specify what was the rationale for selection of the activity setting and target group.
11.	Baseline prevalence of TB in the target population		If not available/known, please mention 'NA'
**Type of ACF**			
12.	House-to-House survey/screening	Yes / No	Whether the house-to-house visit was done to identify the individual with presumptive TB patient?

13.	Health Education	Yes / No	Whether the the health worker provided health education during the visit to identify presumptive TB patient?
14.	Personnel involved in the activity	□ Community health worker/volunteer<br>□ Health worker from general health system<br>□ Field staff recruited for project<br>□ RNTCP staff<br>□ Anganwadi worker<br>□ Other (specify):	Mark multiple boxes if there are multiple answers
15.	Whether personnel were incentivised for the ACF activity? If yes, mention	Yes / No	[amount per day / amount per case]
16.	Target number of houses per day per health worker		How many houses each health worker involved in the ACF was supposed to cover per day?
17.	Who were interviewed by the health workers for identifying presumptive TB patients through symptom screening at households?	□ Each individual were interviewed<br>□ Interviewed the head of the house-hold about all the household members<br>□ Interviewed the available member during the visit about all the household members<br>□ Interviewed only those available during the house visit<br>□ Others:	Please specify how the health workers conducted the interview to identify the presumptive TB patient on reaching the target house
18.	Symptoms included to define the presumptive TB patient for ACF	□ Cough of any duration<br>□ Cough for more than or equal to two weeks<br>□ Fever of any duration<br>□ Fever for more than or equal to two weeks<br>□ Weight loss<br>□ Night sweats<br>□ Hemoptysis<br>□ Others:	Mark multiple boxes if there are multiple answers
19.	Did health worker facilitate the linkage of identified presumptive TB patient to get the investigations done?	Yes / No	Were any additional efforts made to ensure whether the identified presumptive TB patient undergoes the investigations for diagnosis of TB?
20.	How the presumptive TB patients were linked to the investigations?	□ Issued referral letter to get the investigations done at DMC<br>□ Accompanied referral<br>□ Sputum was collected and transported by the health worker<br>□ Travel support was provided to patient<br>□ Vouchers/monetary support to get the chest X-ray<br>□ Any others:	If the answer is 'No' to previous question, please skip this question
21.	Mobile vans / temporary depot for TB screening stationed in the activity area	Yes / No	
22.	Investigations used for diagnosis of TB in presumptive TB patients	□ Only Sputum Smear Microscopy	Please tick the appropriate algorithm followed by the team

		☐ Sputum Smear Microscopy followed by chest x-ray for those with high clinical suspicion<br>☐ Sputum Smear Microscopy and Chest x-ray in parallel<br>☐ Sputum Smear Microscopy and Chest x-ray in parallel followed by GeneXpert when Chest x-ray is positive and sputum smear micrscopy is negative<br>☐ Upfront chest x-ray followed by Sputum Smear Microscopy (SSM) for only those with chest x-ray suggestive of TB<br>☐ Upfront chest Xray followed by GeneXpert<br>☐ GeneXpert and Chest Xray in parallel<br>☐ Only GeneXpert<br>☐ Others:	
23.	Case definition of 'Bacteriologically confirmed TB'		Please provide how someone was labelled as bacteriologically confirmed TB under the project
24.	Case definition of 'Clinically diagnosed/smear negative pulmonary TB'		Please provide how someone was labelled as 'Clinically diagnosed/smear negative pulmonary TB' under the project
25.	Did health worker facilitate the linkage of identified TB patient to avail the treatment?	Yes / No	Any additional efforts made to ensure that the identified TB patient are linked to treatment
26.	Please describe how the health workers supported initiation of treatment		If you have answered 'No' to the previous question, please skip this question
27.	Whether the treatment initiation details were recorded under the project?	Yes / No<br>If yes, details:	Any additional efforts made to record the treatment initiation details of ALL the TB patients identified under the project
28.	Whether the treatment outcome details were recorded under the project?	Yes / No<br>If yes, details:	Any additional efforts made to record the treatment outcomes of ALL the TB patients identified under the project
**Outputs/Outcomes**			
29.	Total number of target population mapped for the activity		This is the total number among whom you intended to conduct the ACF activity. It could be the population of the area you selected. If not available, please mention 'NA'
30.	Number of individuals who underwent symptom screening		
31.	Number of individuals screened positive during the symptom screening		
32.	Number of individuals who underwent Sputum smear microscopy		

33.	Number of individuals who underwent Chest X-ray		
34.	Number of individuals who underwent Genexpert		
35.	Number of individuals diagnosed with pulmonary TB		
36.	Number of patients diagnosed by sputum smear microscopy		
37.	Number of patients diagnosed by Chest X-ray		
38.	Number of patients diagnosed by Genexpert		
39.	Number of patients diagnosed as bacteriologically confirmed pulmonary TB		
40.	Number of patients diagnosed as clinically diagnosed pulmonary TB		
41.	Number of false positive results		Positive on Sputum smear microscopy but negative on Gene Xpert
42.	Number of patients clinically diagnosed with Extra-pulmonary TB cases		
43.	Number of TB patients initiated on treatment		
44.	Number of individuals completing the treatment?		Mention the method used to check adherence, if any
45.	Number of deaths among the patients initiated on treatment		
46.	Number of loss-to-follow up among the patients initiated on treatment		
47.	Any documentation of change in TB case notification rate due to ACF?		
48.	Additional comments: *(Note: If there are any additional comments, regarding implementing the ACF programme or documenting data please feel free to provide more information in additional sheets)*		

Please provide the details of contact person, who can be contacted to get clarification on the above mentioned details:

1. Name:
2. Designation:
3. Organisation:
4. Mobile No:
5. Email id:
