# Supplementary material for: Active Case Finding for Tuberculosis in India: A Syntheses of Activities and Outcomes Reported by the National Tuberculosis Elimination Programme
Source: Trop Med Infect Dis. 2021 Nov 30;6(4):206. doi: 10.3390/tropicalmed6040206 (PMC8705069; doi:10.3390/tropicalmed6040206)
Supplement: Supplementary file 1 [file tropicalmed-06-00206-s001.zip › Supplementary Document S1-Data management and analysis.pdf]

Supplementary Document S1. Data management and analysis.

Data extraction:
One of us (SBN) extracted and collated data from the data proformas provided by the programme managers from the states and implementing partner. PT and SS independently checked the extracted data and all authors reviewed and discussed the data.

The following details were extracted from the data proformas:

Description of ACF activity: Study settings (urban or rural); year-wise duration of activity (number of days); frequency of activity; the basis for selection of area or population; the mapped target population; the baseline prevalence of TB in the target population.

Type of ACF Activity: Whether a house-to-house survey was used; whether health education was provided; whether mobile vans or a temporary depot for TB screening were stationed in the activity area; whether the provider had initiated requests to visit the health facility for screening; the type(s) of personnel involved in the activity; whether personnel were provided incentives for the ACF activity and, if so, the amount or type of incentives provided.

Case definitions and investigations: Case definitions for screening; who were interviewed for symptoms at households; what investigations were used for screening and the algorithm used for screening; the support provided to presumptive TB patients for referral and uptake of screening tests.

Screening outcomes: The target population mapped for the activity; the number of individuals who underwent symptom screening; the number of individuals who screened positive during symptom screening; the number of individuals who underwent diagnostic tests among those positive with symptom screening (Sputum smear, Chest X-ray, GeneXpert); the number of individuals diagnosed with TB; the number of people diagnosed by sputum smear microscopy; the number of people diagnosed by Chest X-ray; the number of patients diagnosed by GeneXpert / CBNAAT (cartridge-based nucleic acid amplification test); the number clinically diagnosed with pulmonary TB; the number of false positive results; and the number clinically diagnosed with extra-pulmonary TB.

TB treatment: The number of TB patients initiated on treatment; the methods used to assess treatment adherence and the outcomes; the number of individuals completing the treatment; the number of deaths among the patients initiated on treatment; the number lost-to-follow up; and any documentation of change in TB case notification rate due to ACF.

We also requested information about any challenges faced during implementing ACF.

Assessment of data quality and completeness
We assessed the overall data quality by enumerating the gaps in the data provided by each state TB programme and by each partner agency in the domains discussed above. We attempted to assess whether these gaps reflected issues related to the collection of data, the format for recording data, the storage of data, or it's retrieval; or resource constraints related in replying to our requests. Since we only had access to whatever data was provided by the NTEP programme managers and partner agencies, we could not assess the actual quality of the processes and procedures used. We recorded the details of the activities described in the proformas and reports provided by the NTEP programme managers and partner agencies about the populations mapped and screened, the personnel involved and incentives provided, the involvement of the personnel in the ACF cascade, the diagnostic algorithms and the case definitions used. From their responses, we recorded the methods used to ensure if the target populations mapped and screened were representative; the proportions screened for TB among the vulnerable population mapped; what screening and confirmatory tests were done and if the numerical data provided for the proportions screened and tested were available in a

detailed spreadsheet format for each screening phase, or only as total numbers without corroborative information. We also looked for discrepancies in the data provided. We assessed if the loss of data was minimal after screening till diagnostic tests were done; if the loss of data was minimal after diagnosis till treatment initiation; and if there was any further follow up. We also noted any additional data provided by each state or partner agency that were considered as innovations or improvements aimed at enhancing screening, referral, case-detection, treatment-initiation, treatment adherence, and treatment completion; or of data management in general. We followed the guidance provided in the TIDieR-PHP reporting guideline for population health and policy interventions to describe these details. We tabulated our assessments of the completeness of reporting key components.

Dealing with missing data

We attempted to obtain missing data/information from programme managers or partner agency contacts. If discrepancies remained in the numbers screened and the numbers reported at each stage of ACF activity, we considered losses-to-follow-up during any stage of the diagnostic cascade as not having successfully completed that stage of the ACF cascade (initiated screening, screening positive, being diagnosed with TB, initiating treatment, completing treatment).

Data Analysis

Since the data were heterogeneous with regard to the ACF activities, the vulnerable groups screened, and the diagnostic tests used, we did not attempt to synthesize data in a meta-analysis, but tabulated our results following guidance provided in the synthesis without meta-analysis (SWiM) guideline [24]. For each year from 2017 to 2019, we recorded for each state and partner agency the available data about the number and proportion of people screened from the mapped target population, identified with presumptive TB, and tested for TB. We recorded the number and types of diagnostic tests done and the proportions diagnosed with each. We derived the total number and proportion of TB cases detected (yield) along with the 95% confidence intervals (CI).

We also tabulated the available summary national ACF data from all states and union territories in India that reported ACF activities to the NTEP from the India TB annual reports for the year 2018 [20], 2019 [21] and 2020 [22]. Detailed summary ACF data for 2017 were not available in the corresponding annual report [19].

We assessed the proportions mapped, screened, identified, tested, and diagnosed (and treated) against the expected proportions set by the NTEP for the ACF programme as detailed in Box 1. We attempted to use this as a framework to evaluate comparative diagnostic yields by looking for possible associations between these proportions and TB detection rates. We also attempted to assess whether the different strategies used to enhance ACF activities (incentives to programme staff or patients) impacted on various steps of the ACF cascade. We derived the number needed to screen (NNS), which is the number of individuals who were screened to identify one person diagnosed with TB (number screened/number diagnosed with TB) for each year for each state and partner agency [25]. We examined the relationship between the proportions completing relevant parts of the ACF cascade and the NNS. We also reported the treatment initiation rates and treatment completion rates, loss to follow up rates, and mortality rates (where available).

To assess the challenges in implementing ACF activities, we used information from the returned data proformas and information gathered from discussions with programme managers and partner agencies. We listed them under the broad themes of challenges related to the health system, healthcare provider and patient-related challenges. We attempted to integrate the challenges according to these themes to evaluate their effects on implementing ACF activities.

Changes in data management between the registered protocol and the review

We also made some changes to the review process after the protocol of this re-view was registered that was necessitated by the data available for evaluation.

The first was in separately reporting the results of the review of published data, from largely non-programme activities, from the programme data directly obtained from the states and union territories, supplemented by summary data from the NTEP annual reports, reviewed herein. We thought this would be appropriate since it permitted a more realistic assessment of ACF, based on programmatic perspectives, rather than in the context of research studies.

The other changes related to some of the methods of the review.

1. We replaced the risk of bias instrument that we had initially intended to use with an assessment of the completeness of reporting, since the former was tailored towards assessing the risk of bias from observational studies. Our current assessment of the completeness of reporting and overall data quality is limited by the data we were provided for the domains assessed.

2. We also changed the frame-work of analysis from what we originally planned in the protocol that was based on the framework provided in a review [10] that was more appropriate for research studies then for programme data. We thought that evaluating the process and outcomes of ACF against the assumptions and expectations of the NTEP would provide more pragmatic information.

3. The NNS was added to the analysis framework since the WHO considers this a useful indicator in monitoring the efficiency of ACF activities [25, 50]. We only calculated the crude NNS for each year for each state based on the total population screened that year divided by the numbers diagnosed with TB. Some states provided data for the numbers screened and diagnosed in every district during each phase of ACF activity. In some states we observed that different areas were mapped for screening in each phase. In other states the data indicated that although the same districts were mapped for different phases in a year, the numbers targeted for screening differed. Ideally, the NNS for each phase weighted by the population size should have been calculated, and the mean NNS weighted by the population size for each ACF phase conducted in a calendar year calculated, and reported for each state. However, this breakdown of data by districts and phases of ACF was not available from most states, so we used the total population screened and the numbers diagnosed for the whole year to derive the NNS. We used the medians and the inter-quartile range to summarize data for NNS across the states.
