# Supplementary material for: Active Case Finding for Tuberculosis in India: A Syntheses of Activities and Outcomes Reported by the National Tuberculosis Elimination Programme
Source: Trop Med Infect Dis. 2021 Nov 30;6(4):206. doi: 10.3390/tropicalmed6040206 (PMC8705069; doi:10.3390/tropicalmed6040206)
Supplement: Supplementary file 1 [file tropicalmed-06-00206-s001.zip › Supplementary Tables S1- S3.pdf]

Supplementary Table S1. Summary of the completeness of reporting of ACF activities.

	ACF rounds		Mapping		Screening					Diagnosis			Treatment		Impact	Data
Implementor	Frequency	Duration	Target Population	Basis for mapping	Persons screened	Personnel	Incentives	Linkage to testing	Screening outcomes	Diagnostic algorithm	Case definitions	Test results	Treatment initiation	Treatment outcomes	Impact on notification	Amount of detail
Andaman & Nicobar	A	A	A	A	A	A	A	PA	A	A	A	A	A	A	NA	PC
Andhra Pradesh	A	A	A	A	A	A	A	A	A	A	A	PA	A	A	NA	YC
Bihar	A	A	A	A	A	A	A	A	PA	A	A	A	NA	NA	NA	PD
Gujarat	A	A	A	A	A	A	PA	A	A	A	A	A	PA	NA	NA	PD
Karnataka	A	A	A	A	A	PA	A	A	PA	A	A	A	NA	NA	NA	PD
Ladakh	A	A	A	A	A	A	A	A	NA	A	A	A	A	A	NA	PC
Maharashtra	A	A	A	A	A	A	A	A	A	A	A	A	A	NA	NA	PD
Manipur	A	A	A	A	A	A	PA	A	A	A	A	A	NA	NA	NA	YC
Mizoram	A	A	A	A	A	A	A	A	A	A	A	A	A	A	NA	YC
Tamil Nadu	A	NA	A	A	A	A	A	A	A	A	A	PA	PA	NA	NA	YC
Uttarakhand	A	A	PA	PA	NA	NA	NA	NA	NA	NA	NA	A	NA	NA	NA	YC
The Union: Axshya	A	A	A	A	A	A	A	A	PA	A	A	A	A	NA	NA	YC
ICMR: TIE-TB	A	A	A	A	PA	PA	NA	A	PA	A	A	A	A	A	A	PD
KHPT: THALI	A	A	A	A	A	A	PA	A	PA	A	A	PA	A	NA	NA	YC
WHP: THALI	A	A	A	A	A	A	A	A	PA	A	A	A	A	NA	NA	YC
WVI: Axshya	A	A	A	A	A	A	PA	A	PA	A	A	PA	A	NA	NA	YC

Evaluation of the adequacy of reporting from returned data-proformas. Key: A = Adequate; PA = Partly adequate; PC = Phase-wise, consolidated; PD = Phase-wise, district-wise; NA = not available; YC = Year/years, consolidated. Abbreviations: ICMR = Indian Council for Medical Research; KHPT = Karnataka Health Promotion Trust; WHP = World Health Partners; WVI = World Vision India.

Supplementary Table S2. Summary data from the National Tuberculosis Elimination Programme for active case finding (ACF) activities in 2018 from states and union territories (ranked by population size) *.

State/Union Territory	Vulnerable Target Population Mapped	Numbers Screened from Mapped Target Population (%)	Numbers with Presumptive TB Tested from Those Screened (%)	TB Diagnosed in Those Tested (%; 95% CI)	Number Needed to Screen (NNS)
Uttar Pradesh	75,435,905	71,302,155 (95)	479,446 (0.7)	21,779 (4.5; 4.4 to 4.6)	3,274
Maharashtra	18,901,687	16,853,553 (89)	61,604 (0.4)	4,143 (6.7; 6.5 to 6.9)	4,068
Bihar	9,747,689	3,148,051 (32)	51,380 (1.6)	2,483 (5.5; 5.4 to 5.7)	1,268
West Bengal	10,857,140	10,021,058 (92)	173,825 (1.7)	1,878 (1.1; 1.0 to 1.1)	5,336
Madhya Pradesh	NA	NA	NA	NA	NA
Tamil Nadu	3,566,777	2,972,314 (83)	357,667 (12.0)	1,528 (0.43; 0.41 to 0.45)	1,945
Rajasthan	2,094,000	1,691,000 (81)	29,161 (1.7)	606 (2.1; 1.9 to 2.3)	2,790
Gujarat	22,410,251	17,880,000 (80)	99,636 (0.6)	4,154 (4.2; 4.1 to 4.3)	4,307
Karnataka	10,347,300	10,265,692 (99)	99,946 (1.0)	2,957 (3.0; 2.9 to 3.1)	3,472
Andhra Pradesh	9,922,758	5,130,826 (52)	35,785 (100)	1,771 (4.7; 4.5 to 4.9)	21
Odisha	4,235,030	1,196,031 (28)	42,133 (3.5)	939 (2.2; 2.1 to 2.4)	1,274
Jharkhand	3,723,237	3,132,870 (84)	14,013 (0.5)	1,095 (7.8; 7.4 to 8.3)	2,861
Telangana	3,01,509	54,861 (18)	2,594 (5.4)	106 (4.1; 3.4 to 4.9)	517
Assam	353,946	133,181 (38)	7,114 (5.3)	547 (7.7; 7.1 to 8.3)	243
Kerala	34,100,000	27,662,028 (81)	26,487 (0.1)	329 (1.2; 1.1 to 1.4)	84,079
Punjab	6,235,317	798,500 (13)	7,217 (0.9)	481 (6.7; 6.1 to 7.3)	1,660
Chhattisgarh	29,800,000	13,177,785 (44)	14,648 (0.1)	986 (6.7; 6.3 to 7.2)	13,364
Haryana	3,688,846	1,534,024 (42)	15,474 (1.0)	548 (3.5; 3.3 to 3.8)	2,799
Delhi	15,000	4,900 (33)	324 (6.6)	15 (4.6; 2.8 to 7.5)	327
Jammu & Kashmir	175,378	75,834 (43)	3,830 (5.1)	187 (4.9; 4.2 to 5.6)	406
Uttarakhand	1,343,500	561,649 (42)	3,658 (0.7)	270 (7.4; 6.6 to 8.3)	2080
Himachal Pradesh	954,658	925,284 (97)	11,398 (1.2)	315 (2.8; 2.5 to 3.1)	2937
Tripura	NA	NA	NA	NA	NA
Meghalaya	2,36,421	148,521 (63)	705 (0.5)	20 (2.8; 1.8 to 4.3)	7426
Manipur	NA	NA	NA	NA	NA
Nagaland	86,501	11,343 (13)	1,587 (14.0)	56 (3.5; 2.7 to 4.6)	202

Arunachal Pradesh	92,215	5,040 (6)	1,535 (30.5)	27 (1.8; 1.2 to 2.6)	186
Goa	120,000	63,000 (53)	825 (1.3)	18 (2.2; 1.4 to 3.4)	3,500
Puducherry	954	953 (100)	50 (5.3)	0 (0.0)	NA
Mizoram	5,113	4,186 (82)	434 (10.4)	28 (6.5; 4.5 to 9.2)	150
Chandigarh	2,56,000	9,500 (4)	13 (0.1)	6 (46.2; 23.2 to 70.9)	1,583
Dadra & Nagar Haveli and Daman & Diu	460,000	320,000 (70)	723 (0.2)	18 (2.5; 1.6 to 3.9)	17,778
Sikkim	4,779	1,083 (23)	63 (5.8)	2 (3.2; 0.9 to 10.9)	542
Andaman & Nicobar	51,203	3,136 (6)	103 (3.3)	6 (5.8; 2.7 to 12.1)	522
Lakshadweep	69,142	65,482 (95)	183 (0.3)	2 (1.1; 0.3 to 3.9)	32,741
**India**	**249,939,256**	**189,364,840 (75.7)**	**1,543,860 (0.8)**	**47,307 (3.1; 3.0 to 3.1)**	**Median: 2080 (IQR 517 to 4068)**

*Modified from annexure 1.7 in the India TB report- 2019 [20]. Madhya Pradesh merged ACF activity with a state initiative to detect TB, Leprosy and non-communicable diseases through house-to-house surveys conducted in two blocks of every district twice a year. Manipur & Tripura did not conduct ACF in 2018. CI = Confidence interval; IQR = Interquartile range.

Supplementary Table S3. Summary data from the National Tuberculosis Elimination Programme for active case finding (ACF) activities in 2019 from states and union territories (ranked by population size) *.

State/Union Territory	Vulnerable Target Population Mapped	Numbers Screened from Mapped Target Population	Numbers with Presumptive TB Tested from Those Screened (%)	TB Diagnosed in Those Tested (%; 95% CI)	Number Needed to Screen (NNS)
Uttar Pradesh	71,011,915	68,429,974 (96)	428,042 (0.6)	20,807 (4.9; 4.8 to 4.9)	3,288
Maharashtra	95,163,760	87,568,441 (92)	211,750 (0.2)	9,737 (4.6; 4.5 to 4.7)	8,993
Bihar	29,500,000	10,185,550 (35)	14,252 (0.1)	709 (5.0; 4.6 to 5.3)	14,366
West Bengal	16,886,918	15,756,571 (93)	300,103 (1.9)	2,657 (0.9; 0.8 to 0.9)	5,930
Madhya Pradesh	NA	NA	NA	NA	NA
Tamil Nadu	7,436,669	2,680,756 (36)	40,703 (1.5)	2,542 (6.3; 6.0 to 6.5)	1,054
Rajasthan	14,822,610	10,551,160 (71)	85,159 (0.8)	3,243 (3.8; 3.7 to 3.9)	3,254
Gujarat	20,469,660	15,833,566 (77)	90,318 (0.6)	1,998 (2.2; 2.1 to 2.3)	7,924
Karnataka	19,335,483	299,157 (2)	253,601 (84.8)	4,841 (1.9; 1.8 to 2.0)	62
Andhra Pradesh	50,383	34,923 (69)	1,035 (3.0)	25 (2.4; 1.6 to 3.5)	1396
Odisha	5,792,098	2,210,783 (38)	64,045 (2.9)	1,272 (2.0; 1.9 to 2.0)	1,738
Jharkhand	55,720,950	44,920,834 (81)	34,147 (0.1)	3,902 (11.4; 11.1 to 11.8)	11,512
Telangana	7,560,886	2,328,711 (24)	31,583 (1.4)	2,117 (6.7; 6.4 to 7.0)	1,100
Assam	187,472	44,886 (24)	4,212 (9.4)	190 (4.5; 3.9 to 5.2)	236
Kerala	775,000	133,949 (17)	2,274 (1.7)	47 (2.1; 1.6 to 2.7)	2,850
Punjab	3,612,160	2,392,039 (66)	10,319 (0.4)	312 (3.0; 2.7 to 3.4)	7,667
Chhattisgarh	29,500,000	10,185,550 (35)	14,252 (0.1)	709 (5.0; 4.6 to 5.3)	14,366
Haryana	13,324,275	1,568,642 (12)	12,763 (0.8)	215 (1.7; 1.5 to 1.9)	7,296
Delhi	4,57,453	2,47,669 (54)	6,122 (2.5)	467 (7.6; 7.0 to 8.3)	530
Jammu & Kashmir	1,208,111	93,010 (8)	3,667 (3.9)	141 (3.9; 3.3 to 4.5)	659
Uttarakhand	248,771	177,699 (71)	2,433 (1.4)	72 (3.0; 2.4 to 3.7)	2,468
Himachal Pradesh	1,817,691	1,813,396 (100)	41,643 (2.3)	1,449 (3.5; 3.3 to 3.7)	1,251
Tripura	773,231	55,808 (7)	55,808 (100.0%)	84 (0.2%; 0.1 to 0.2)	664
Meghalaya	125,103	57,683 (46)	997 (1.7)	16 (1.6; 1.0 to 2.6)	3,605
Manipur	NA	NA	NA	NA	NA
Nagaland	21,179	11,151 (53)	1,061 (9.5)	40 (3.8; 2.8 to 5.1)	279

Arunachal Pradesh	50,383	34,923 (69)	1,035 (3.0)	25 (2.4; 1.6 to 3.5)	1,397
Goa	102,563	83,621 (82)	314 (0.4)	1 (0.3; 0.1 to 0.2)	8,3621
Puducherry	46,709	46,709 (100)	245 (0.5)	4 (1.6; 0.6 to 4.1)	11,677
Mizoram	29,298	22,304 (76)	573 (2.6)	12 (2.1; 1.2 to 3.6)	1,859
Chandigarh	NA	NA	NA	NA	NA
Dadra & Nagar Haveli and Daman & Diu	322,434	270,290 (84)	294 (0.1)	8 (2.7; 1.4 to 5.3)	33786
Sikkim	43,817	20,044 (46)	689 (3.4)	12 (1.7; 1.0 to 3.0)	1670
Andaman & Nicobar	50,000	5,990 (12)	490 (8.2)	86 (17.6; 14.4 to 21.1)	70
Lakshadweep	70,177	0	NA	NA	NA
**India**	**39,27,38,350**	**27,73,58,984 (70.6)**	**20,46,453 (0.7)**	**62,958 (3.1%; 3.0 to 3.1)**	**Median: 2468 (IQR = 1050 to 7924)**

*Modified from annexure 6 in the India TB report- 2020 [21]. Chandigarh, Madhya Pradesh & Manipur did not conduct separate ACF activities.
